# Evaluation of a technical advisory board for an occupational injury surveillance research project: A qualitative study

**DOI:** 10.1002/hsr2.777

**Published:** 2022-08-08

**Authors:** Amelia Vaughan, Viktor Bovbjerg, Solaiman Doza, Laurel Kincl

**Affiliations:** ^1^ College of Public Health and Human Sciences Oregon State University Corvallis Oregon USA

**Keywords:** advisory board, commercial fishing, occupational injury, safety, surveillance

## Abstract

**Background and Aims:**

Advisory boards play a key role in guiding and informing research programs, including occupational health surveillance. It is important to evaluate the effectiveness of these advisory boards. This report details the organization of the Risk Information System for Commercial (RISC) Fishing Technical Advisory Board (TAB), the approach taken to evaluate the TAB, and the results of the evaluation. The RISC TAB was formed to provide advice and recommendations to the study team and informed the development and use of the safety surveillance system.

**Methods:**

The evaluation approach was informed by limited previous literature on advisory board assessments. This evaluation was conducted in Year 5 of the 6‐year project. A review of the meeting notes, materials and correspondences, and study progress was conducted internally to document input from the board and associated actions. To obtain member perspectives, we surveyed the TAB and discussed it in a subsequent TAB meeting.

**Results:**

The RISC Fishing TAB members constitute a wide variety of commercial fishing safety stakeholders. The internal analysis identified the main project aspects and 14 of the proposed changes from the TAB that have either been implemented or are in progress in the project. Ten of the 15 TAB members responded indicating a positive experience on board organization and conduct.

**Conclusion:**

Evaluation of advisory boards is an essential part of a research program. A process is outlined in this report to inform future efforts to document measurable ways to inform projects based on advisory board feedback and reflections.

## 1 INTRODUCTION

Scientific advisory boards are common in private and public organizations and are mandated in federal agencies.^1^ Advisory boards also play a key role in guiding and informing research programs. External advisory boards for occupational safety and health (OSH) surveillance are used in the National Institute for Occupational Safety and Health (NIOSH) state‐based surveillance programs. This report describes an evaluation of the effectiveness and impact of a technical advisory board (TAB) established to support the formation and activities of the Risk Information System for Commercial (RISC) Fishing, an OSH surveillance research project. RISC Fishing is a recently developed, comprehensive commercial fishing injury surveillance system that utilizes data from US Coast Guard (USCG) injury reports, state and national Trauma Registries, and Emergency Medical Service (EMS) data to estimate injury risk and risk factors in commercial fishing in the Pacific Northwest (PNW). RISC Fishing aims to provide a resource for PNW commercial fisheries to develop, evaluate, and inform safety initiatives by combining these sources of data into an ongoing, scalable, adaptable surveillance system and to trial the use of the system for hazard assessments and evaluations of safety and fishery management interventions. Previous publications have outlined both the process of matching data^2^ and the effort to explore publicly available charts to obtain commercial fishing‐specific estimates of workplace exposures, safety behaviors, health status, illnesses, and injuries, as well as working and employment conditions.^3^

As noted in the Center for Disease Control's Updated Guidelines for Evaluating Public Health Surveillance Systems, it is important that surveillance systems and advisory boards that oversee them are evaluated regularly.^4^ Many private entities, nonprofit organizations, and academic institutions operate advisory boards but little research has been done on the impact of advisory boards and how to evaluate them. A recent effort to improve the evaluation of OSH surveillance systems has been undertaken,^5^ but did not specifically address advisory board evaluations. Some literature was available describing an evaluation of community health‐based advisory boards and private entity advisory boards.^6^, ^7^ We found no published research looking at scientific advisory board members' level of satisfaction or a framework for evaluating the advisory boards they serve on.

The RISC Fishing TAB (RISC TAB) was established in 2017 and has met biannually throughout the project period (5 years to date). This report details the operation of the RISC TAB, the approach taken to evaluate the TAB, and the results of the evaluation. Our report can help to improve the utilization of future advisory boards that will benefit OSH research projects.

## 2 METHODS

The purpose of the RISC TAB is to identify relevant commercial fishing industry issues and priorities and to help translate surveillance findings into tailored, nimble responses for hazard assessment needs of important fishery safety stakeholders. The RISC TAB provides advice and recommendations to the study team and informed the development and use of the safety surveillance system. Project‐specific topics shared with the TAB included the: (1) database architecture; (2) data elements and coding; (3) data entry and query interfaces; (4) reporting elements; (5) report contents and data visualization; (6) priorities for hazard assessments; and (7) dissemination materials and methods (e.g., tutorials, hazard sheets).

RISC TAB members were recruited based on their interest, ability to represent key stakeholders, and availability to commit time to the project. As this project is currently restricted to Oregon and Washington, all members represented commercial fishing interests in those states. The RISC TAB members constitute a wide variety of commercial fishing safety stakeholders (including state and national government agencies), public health surveillance practitioners, and OSH researchers and practitioners (Table 1).

**Table 1:** RISC Fishing TAB members

Researchers and students from Oregon State University and the University of Washington
Oregon and Washington Sea Grant Commercial Fisheries Extension Agents
Fishing community members
Washington and Oregon Departments of Fish and Wildlife fisheries management specialists.
Researchers at the National Institute of Occupational Safety and Health, Pacific Northwest Agricultural Safety and Health Center, and the Northeast Center for Occupational Health and Safety
Fishing safety practitioners from the Alaska Marine Safety and Education Association and American Seafoods
NOAA fisheries economists

Abbreviations: NOAA, National Oceanic and Atmospheric Administration; RISC, Risk Information System for Commercial; TAB, Technical Advisory Board.

To date, nine semiannual meetings have been held over the 5 years of the project with meetings occurring both virtually and in person. During the pandemic, all meetings were virtual, but because the TAB was already well established, it continued to function effectively. Meetings were conducted in a professional but comfortable manner and facilitated by the project Principal Investigators. At the onset, TAB expectations were set with appropriate ground rules (see Table 2).^8^ Agendas, including specific premeeting actions for board members, were shared ahead of time, and expectations were set with appropriate ground rules, and meeting notes/decisions/action items circulated regularly. Approval of activities by an Institutional Review Board was determined to not be necessary due.

**Table 2:** RISC Fishing TAB ground rules

1. Stick to agenda topics.
2. Keep the discussion focused on one subject at a time.
3. Discuss all relevant information and issues, even difficult ones.
4. Keep the discussion open and balanced.
5. Speak for yourself, not for others in the room.
6. Actively listen.
7. Avoid repetition.
8. Be respectful of others.
9. Disagree openly, but try not to be disagreeable.
10. Look for mutually beneficial solutions.
11. Contribute to the discussion.
12. Follow through on commitments.

Abbreviations: RISC, Risk Information System for Commercial; TAB, Technical Advisory Board.

### 2.1 RISC TAB EVALUATION

To determine the TAB's effectiveness and impact, we analyzed meeting notes and surveyed the TAB members in Year 5. The purpose of the evaluation was to both assess the impact of the TAB on the research project as well as the level of satisfaction and impact of the TAB members in serving on the board as it related to their own work. This report also details the lessons learned and best practices for facilitating an effective and impactful scientific advisory board.

The research team first reviewed the TAB members and retention over the project. Then, a detailed review was undertaken of the meeting notes, presentations, shared materials, and email correspondences from the nine RISC TAB meetings to evaluate feedback from the board and what ideas were incorporated into the research project and development and application of the RISC Fishing system and outreach materials. This review provided a record over the project time period of both feedback and idea implementation that could be compared. To complete this comparison, themes discussed regarding the aspect of the project were identified, feedback summarized, and project direction assessed.

A survey was developed to ask TAB members to provide feedback on their experience serving on the board and to give constructive ideas on how to improve TAB facilitation and stakeholder representation. The survey was distributed via Qualtrics to the current 15 TAB members in August of 2021, before our most recent meeting. During the meeting, we reviewed the results of our evaluation of the meeting notes and explained the importance of the TAB survey, and presented the survey with the meeting notes. The survey questions were first drafted by the lead author and revised by the research team and the PNW Agricultural Safety and Health (PNASH) Center's outreach core.^9^ The initial draft of the survey contained a section on a self‐evaluation which was removed to keep the survey brief and most relevant. Table 3 shows the final survey.

**Table 3:** RISC Fishing Advisory Board Survey

Likert scale questions (5‐point scale: Strongly agree to Strongly disagree)
1. Advisory board members are knowledgeable about commercial fishing needs and the issues that the industry face.
2. Advisory board meetings are productive.
‐ If you disagree, please share with us any concerns and ideas you have.
3. There are a sufficient number of advisory board meetings throughout the year.
‐ If not, how many do you recommend?
4. The quality, quantity, and timing of the information given to advisory board members is adequate.
‐ If you disagree, please share with us any concerns and ideas you have.
5. The agendas of our meetings and supporting written material are provided in advance of meetings.
6. Board meetings are generally well‐run and make good use of members' time.
‐ If you disagree, please share with us any concerns and ideas you have.
7. Our board's size is about right.
‐ If you disagree, please share with us what size and composition could have worked better.
General open‐ended questions
8. What groups or expertise do you think are missing from this advisory board?
9. What did you enjoy about being on the RISC Fishing Advisory Board?
10. Did you feel your feedback was incorporated into the project's efforts?
11. Did the advisory board help you in your own work?
12. In your opinion what could improve your experience serving on an advisory board?
13. Do you know of any unexpected outcomes? For example, ways the RISC project and/or RISC Technical Advisory Board contributed to any new initiative, partnership, or activity? If so, please describe.
14. What unaddressed or new fishing safety needs and ideas do you have, as we plan future projects at OSU/PNASH?
15. Anything else you would like to tell us?
Demographics information
16. Name:
17. Organization:
18. Position:
19. How long have you been in your current position?
20. How long have you served on the RISC Fishing Advisory Board?
21. Finally, would you be willing to serve on an advisory board again for subsequent OSU/PNASH Commercial Fishing Safety and Surveillance projects?

Abbreviations: OSU, Oregon State University; PNASH, Pacific Northwest Agricultural Safety and Health; RISC, Risk Information System for Commercial.

## 3 RESULTS

At the time of the evaluation, the RISC TAB had 13 of the original 16 members serving continuously since 2017 with a retention rate of 83%. All current board members have been attending meetings for at least the last 3 years.

Analysis of meeting notes, materials, and correspondences identified six main project aspects that were presented at advisory board meetings: (1) Data sources; (2) Database variables; (3) Data coding; (4) Advisory Board Members; (5) RISC Fishing System Tool; and (6) Project Outreach and Deliverables. From this the research team was able to track what was presented at each meeting, the feedback collected, and if/how changes were implemented based on the feedback given by TAB members. Fourteen of the proposed changes have either been implemented or are in progress including the addition of EMS data, broadening the scope of searches in state trauma registries to identify more cases for inclusion, adding additional variables, and strategies for stakeholder engagement with the RISC Fishing System tool as well as outreach.

Ten of the 15 TAB members responded to the survey with a response rate of 67%. Respondents included a fishing community member, safety professionals from Alaska Marine Safety Education Association and American Seafood Company, Fisheries Extension Faculty from Oregon State University/Oregon (OSU/OR) Sea Grant, a fisheries economist from the National Oceanic and Atmospheric Administration (NOAA), epidemiologists from NIOSH, safety researchers from PNASH, and a Coastal Shellfish Manager from Washington Department of Fish and Wildlife. Respondents have been in their current position anywhere from 2 to 24 years with an average of 10.8 years. Seven respondents indicated that they would be willing to serve on the advisory board again for subsequent OSU/PNASH Commercial Fishing Safety and Surveillance projects, one said “maybe” and two left the question unanswered. The TAB agreed with or was neutral with every statement in the Likert scale evaluation (see Table 4).

**Table 4:** Responses (*n* = 10) to Technical Advisory Board survey Likert scale question (count)

#	Question	Strongly agree	Agree	Neutral	Disagree	Strongly disagree
1	Advisory board members are knowledgeable about commercial fishing needs and the issues that the industry faces.	10	0	0	0	0
2	Our advisory board's size is about right.	10	0	0	0	0
3	There are a sufficient number of advisory board meetings throughout the year.	9	1	0	0	0
4	Advisory board meetings are productive.	8	2	0	0	0
5	Board meetings are generally well‐run and make good use of member's time.	7	1	2	0	0
6	The agendas of our advisory board meetings and supporting written material are provided an inadequate advance of meetings.	9	1	0	0	0
7	The quality and quantity of the information given to advisory board members are adequate.	10	0	0	0	0

The open‐ended questions generated thoughtful responses. While two board members said the board was not missing stakeholders and it was well rounded, there were suggestions of additional groups/expertise that included an emergency physician who works in a commercial fishing community (*n* = 1) and fishing community members (*n* = 3). More participation from the USCG was mentioned by one advisory board member.

When asked about what the board enjoyed about being on the advisory, a common theme found in every (*n* = 10) response was networking, sharing ideas, respective work in health and safety with other board members, and “making a connection with others working in the area.” One additional concept in response was related to the updates on the progress of the project and “having the opportunity to voice support and provide advice.”

Almost all of the board members (*n* = 9, with 1 nonresponse) indicated they felt their feedback was incorporated into the project. “I feel that the chair does a good job of soliciting input and including it in the design and implementation of projects.” Most board members (*n* = 7, with 3 nonresponses) thought serving on the board did not directly help in their own work but that the collaborations and connections of the board members did help them.

Three board members said they had no specific improvements to suggest. One stated, “this has been one of the better‐managed boards.” We had three in‐person board meetings starting in July 2017 (first meeting) until …… and moved all meetings to be virtual in the Summer of 2020. Four board members cited that more in‐person meetings would have improved their experience, but acknowledged it was not possible with COVID.

Four board members responded to our question if there was an unexpected outcome of their involvement in the advisory board. One stated that nothing came to mind, and two mentioned the Fishermen First Aid and Safety Training, a commercial fishing‐specific training that was developed with the help of injury statistics taken from the RISC Fishing project.^10^ One stated that “this board has emphasized for me the role [that] agency rules can play in fleet safety.”

Six board members provided ideas for unaddressed or new fishing safety ideas for future projects. This included:

- ergonomics of captain's seats and operations,
- development of commercial fishing industry tools to share solutions and strategies across the PNW,
- research into how the insurance could influence fishermen's health and safety,
- what key drivers of risk‐taking such as weather, bar closings, season‐opening, and season timing could have to alleviate risk,
- sleep deprivation,
- hearing loss,
- boatyard hazards,
- other fishermen's health issues,
- safety management systems for small fishing vessels.

While extensive notes were taken at all TAB meetings, they did not necessarily capture feedback from board members in a way that could inform the future evaluation. Tracking changes to the project based on TAB member feedback would be more straightforward if an evaluation form and feedback processes were built into the TAB meetings. Future projects with advisory boards should take this into consideration and provide opportunities for board members to contribute directly to the written record through surveying and a more formal evaluation process.

## 4 CONCLUSION

By conducting our technical advisory board evaluation internally with the notes, materials, and correspondence review, we were able to readily see when feedback helped to shape our project. Externally, by conducting the survey of the TAB members, we learned directly from them what was effective for their engagement as well as opportunities for improvements and future research directions.

The consistency of the TAB members has allowed for rich discussions and feedback and provides the research team with an invaluable sounding board who is well versed in the particulars of the RISC Fishing project.

The internal review shows that the TAB members are an invaluable resource and have greatly contributed to the direction of the project as well as provided insight into their fields and how the RISC Fishing system could be useful to a variety of stakeholders. The survey of TAB members also indicated that they felt that their participation was useful and valuable in forming connections with other stakeholders and learning about what is happening in terms of commercial fishing safety in other fields.

Though advisory boards are common, not much focus has previously been put on formally evaluating their effectiveness. Using the process outlined in this report as a template for future advisory board evaluations could lead to unintended positive outcomes and guide the process to be more focused on measurable ways to inform projects based on TAB feedback and reflections.

## AUTHOR CONTRIBUTIONS

**Amelia Vaughan**: Conceptualization; data curation; formal analysis; methodology; project administration; visualization; writing—original draft. **Viktor Bovbjerg**: Conceptualization; validation; writing—review and editing. **Solaiman Doza**: Writing—review and editing.  **Laurel Kincl**: Conceptualization; formal analysis; funding acquisition; methodology; project administration; supervision; validation; writing—review and editing. All authors have read and approved the final version of the manuscript. Amelia Vaughan had full access to all of the data in this study and takes complete responsibility for the integrity of the data and the accuracy of the data analysis.

## CONFLICT OF INTEREST

The authors declare no conflict of interest.

## TRANSPARENCY STATEMENT

This manuscript is an honest, accurate, and transparent account of the study being reported; no important aspects of the study have been omitted, and any discrepancies from the study as planned (and, if relevant, registered) have been explained.

## Data Availability

The data sets used and/or analyzed during the current study are available from the corresponding author on reasonable request. Amelia Vaughan accepts full responsibility for the accuracy and integrity of the data provided.
